# Nucleation Behavior of a Single Al-20Si Particle Rapidly Solidified in a Fast Scanning Calorimeter

**DOI:** 10.3390/ma14112920

**Published:** 2021-05-28

**Authors:** Qin Peng, Bin Yang, Benjamin Milkereit, Dongmei Liu, Armin Springer, Markus Rettenmayr, Christoph Schick, Olaf Keßler

**Affiliations:** 1 Materials Science, Faculty of Mechanical Engineering and Marine Technology, University of Rostock, Justus-von-Liebig-Weg 2, 18059 Rostock, Germany; qin.peng@uni-rostock.de (Q.P.); benjamin.milkereit@uni-rostock.de (B.M.); olaf.kessler@uni-rostock.de (O.K.); 2 Competence Centre °CALOR, Department Life, Light & Matter, University of Rostock, Albert-Einstein-Str. 25, 18059 Rostock, Germany; christoph.schick@uni-rostock.de; 3 Otto Schott Institute of Materials Research, Friedrich-Schiller-University Jena, Löbdergraben 32, 07743 Jena, Germany; dongmei.liu@uni-jena.de (D.L.); m.rettenmayr@uni-jena.de (M.R.); 4 Medical Biology and Electron Microscopy Centre, University Medicine Rostock, Strempelstr. 14, 18057 Rostock, Germany; armin.springer@med.uni-rostock.de; 5 Institute of Physics, University of Rostock, Albert-Einstein-Str. 23–24, 18059 Rostock, Germany; 6 Butlerov Institute of Chemistry, Kazan Federal University, 18 Kremlyovskaya Street, 420008 Kazan, Russia

**Keywords:** nucleation kinetics, rapid solidification, differential fast scanning calorimetry (DFSC), Al-20Si particle

## Abstract

Understanding the rapid solidification behavior characteristics, nucleation undercooling, and nucleation mechanism is important for modifying the microstructures and properties of metal alloys. In order to investigate the rapid solidification behavior in-situ, accurate measurements of nucleation undercooling and cooling rate are required in most rapid solidification processes, e.g., in additive manufacturing (AM). In this study, differential fast scanning calorimetry (DFSC) was applied to investigate the nucleation kinetics in a single micro-sized Al-20Si (mass%) particle under a controlled cooling rate of 5000 K/s. The nucleation rates of primary Si and secondary *α*-Al phases were calculated by a statistical analysis of 300 identical melting/solidification experiments. Applying a model based on the classical nucleation theory (CNT) together with available thermodynamic data, two different heterogeneous nucleation mechanisms of primary Si and secondary *α*-Al were proposed, i.e., surface heterogeneous nucleation for primary Si and interface heterogenous nucleation for secondary *α*-Al. The present study introduces a practical method for a detailed investigation of rapid solidification behavior of metal particles to distinguish surface and interface nucleation.

## 1. Introduction

In the production chains of metal components, e.g., in spray forming, rapid solidification of metal particles is a critical process. Recently, further interest has been gained in rapid solidification as an important step in additive manufacturing (AM) [1,2,3,4,5]. As important engineering materials, aluminum-based alloys, e.g., Al-Si alloys, are frequently used in AM [6,7,8,9]. In AM processes, the cooling rates are typically from ca. 100 K/s up to 10^6^ K/s [5,10,11,12,13]. For example, in laser assisted powder bed fusion (LPBF), the cooling rate of Al-12Si (mass%) alloy was estimated via simulations, which was above 10^3^ K/s for most parts of the melt pool [11].

Undercooling and nucleation in solidification determine many important aspects of the relevant microstructural features, e.g., the grain size, and the final material properties [14,15,16,17]. Studies regarding the nucleation undercooling of Al-Si alloy particles during rapid solidification processes has been reported [15,18,19,20,21]. However, in-situ observation of the rapid solidification behavior of metal particles is not easily available due to limitations of the measurement capability. For an accurate description of the microstructure evolution during rapid solidification, the nucleation undercooling and nucleation mechanisms are crucial, as they provide access to the optimization of the process parameters. Moreover, accurate measurements of nucleation undercooling and cooling rate, as well as accurate descriptions of rapid solidification mechanisms are required for a better understanding of rapid solidification processes of metal particles at high cooling rates.

In this study, the rapid solidification of Al-20Si (mass%) alloy was investigated by differential fast scanning calorimetry (DFSC) [22] at 5000 K/s cooling, which is in the range of the cooling rate in AM. Micro-sized Al-20Si particles with a diameter of 25 μm were used. A series of 300 nucleation events (300 heating–cooling cycles) were observed using DFSC, and the nucleation kinetics in a single particle was studied by statistical analysis. By combining microstructure and DFSC investigation, the nucleation mechanisms of the primary Si and secondary *α*-Al phases in a rapidly solidified single Al-20Si particle is discussed. Assuming surface and interface nucleation in a spherical cavity for Si and *α*-Al respectively, a modified theoretical model based on classical nucleation theory (CNT) is developed.

## 2. Materials and Methods

In the present work, gas-atomized Al-20Si (mass%) powder particles were used, which is purchased from Nanoval GmbH & Co. KG (Berlin, Germany). The mass fractions of alloying elements of the gas-atomized powder particles was analyzed by X-ray fluorescence spectroscopy (XRF, Bruker, Billerica, MA, USA). The composition is given in Table 1, which is close to the nominal composition.

**Figure 1:**
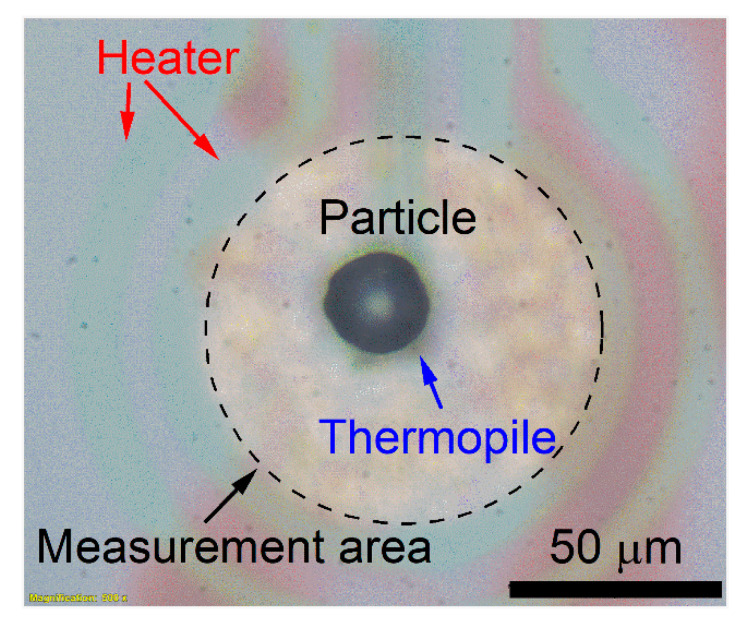
Single Al-20Si powder particle (Particle I) on a UFH1 DFSC sensor.

DFSC based on the thin film sensor (UFH1, Mettler Toledo, Greifensee, Switzerland) was used to characterize the melting and solidification temperatures in-situ. As indicated in Figure 1, film-thermopiles and resistive film-heaters are located at the center of the sensor membrane (amorphous silicon-nitride membrane). One particle (Particle I) of near-spherical shape with a diameter of 25 ± 0.5 µm was chosen and deposited in the center of the measurement area (with a diameter of 90 µm) by a thin copper wire with the help of a stereomicroscope (SZX16, Olympus, Tokyo, Japan) for DFSC measurements, as shown in Figure 1. Before and after the measurements, the particle size was characterized by optical microscopy (Olympus BX41). In order to improve the heat contact between the sensor membrane and the sample, a tiny amount of silicone oil was used. Due to the high heating rate (5000 K/s) and consequent short times (less than 0.12 s) at high temperatures, it was expected that the silicone oil was stable up to high temperatures, even though the boiling temperature of silicone oil is about 250 °C at low heating rates. After the first melting/solidification cycle, a slight flattening of the particle occurred. Thus, improved thermal contact between the sensor membrane and the sample was established. Because of a static temperature offset and a dynamic temperature offset due to thermal lag, the temperature calibration and correction were performed by the eutectic temperature (577 °C) of the Al-Si alloy upon heating at different heating rates before the DFSC measurements. Details of temperature calibration and correction can be found in [23]. For the investigation of the rapid solidification of this particle (Particle I) by DFSC, the particle was heated from 37 °C to 777 °C at a rate of 5000 K/s and held for 0.01 s. This maximum heating temperature ensures a certain overheating at the high heating rate, which is ca. 93 °C higher than the liquidus temperature of Al-20Si (ca. 684 °C [24]). Afterward, the particle was quenched to 37 °C at the same cooling rate. All the DFSC measurements were performed under argon (100 kPa constant pressure).

After rapid solidification by DFSC at 5000 K/s cooling, the particle (Particle I) was transferred from the sensor membrane into an epoxy block. After careful grinding and polishing of the epoxy block, a cross-section of the particle was obtained. After that, the specimen was etched with Weck’s reagent for 4 s [25]. Details of the specimen preparation of one single micro-sized metallic particle for microstructure characterization can be found in [26]. Scanning electron microscopy (SEM) (Zeiss Merlin VP Compact, Oberkochen, Germany) was used to characterize the microstructure (cross-section) of the particle.

To fully characterize the solidification behavior of the particle undergoing rapid solidification at 5000 K/s, another particle (Particle II) with the same size (25 ± 0.5 µm in diameter) as the particle for microstructure characterization (Particle I) was heated and cooled with the same time–temperature profile for 300 times. Note that the temperature calibration and correction were performed before these repeated heating–cooling measurements. Then, the nucleation undercooling, Δ*T*, for each scan was calculated by using the following equation:

(1) $$\Delta T=T_{l}-T_{s\_onset}^{correct}$$

where *T_l_* is the liquidus temperature and $T_{s\_onset}^{correct}$ is the solidification onset temperature (nucleation temperature). The evaluation of $T_{s\_onset}^{correct}$ can be found in [23].

## 3. Results and Discussion

**Figure 2:**
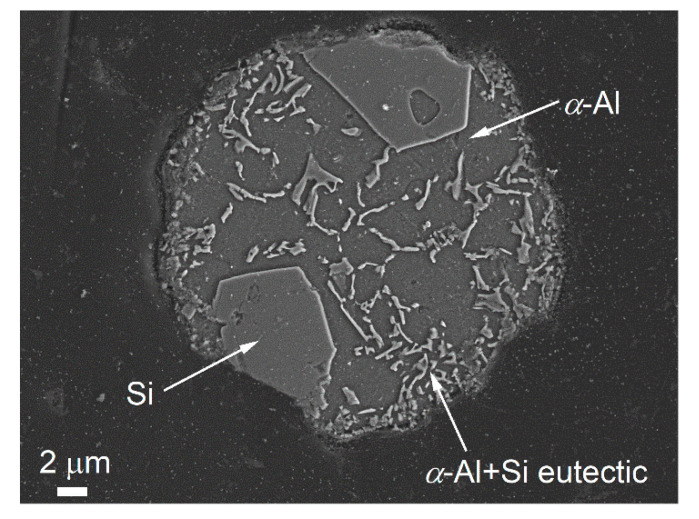
SEM images (secondary electron images) of the cross-section of an Al-20Si particle (Particle I) solidified at 5000 K/s, etched with Weck’s reagent.

The microstructure of the cross-section of a single Al-20Si particle (Particle I) rapidly solidified at 5000 K/s cooling by DFSC was characterized by SEM, as shown in Figure 2. The grey and bright areas in Figure 2 are Al and Si, respectively. The microstructure is comprised of the primary Si, secondary *α*-Al dendrites, and (*α*-Al + Si) eutectics. It seems very likely that the primary Si with polygonal morphology solidified from the surface of the particle (see Figure 2). Unlike the microstructure of gas-atomized particles, only two Si grains were observed because of the lower cooling rate than that of gas-atomized particles.

**Figure 3:**
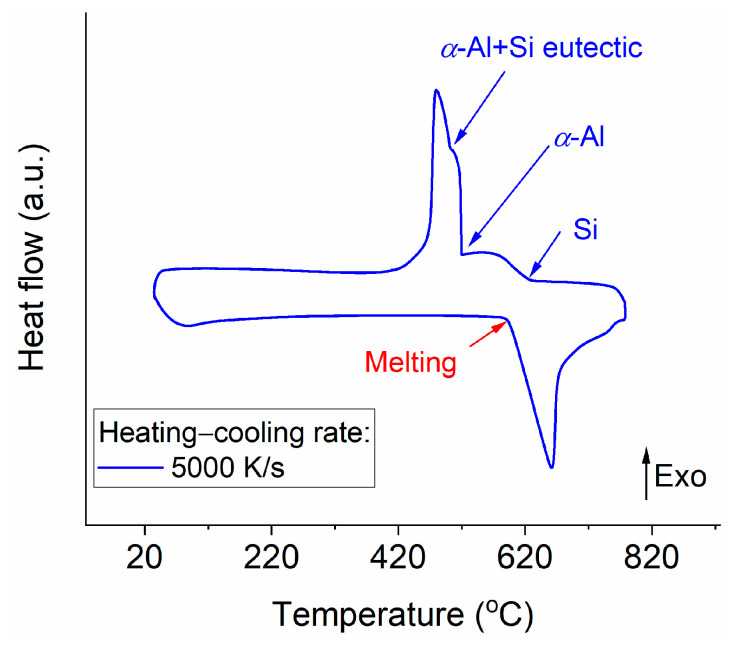
DFSC heat flow curve of a single Al-20Si particle (Particle I) at the cooling rate of 5000 K/s. The melting and solidification onset temperatures of different phases are indicated by arrows.

Figure 3 shows the corresponding DFSC curve at the heating–cooling rate of 5000 K/s for Particle I shown in Figure 2. The primary Si phase starts to solidify first during rapid solidification of an Al-20Si alloy, followed by the formation of the *α*-Al phase, and then the (*α*-Al + Si) eutectic. Two solidification onsets upon cooling (as indicated by the arrows in Figure 3) correspond to the starts of the nucleation of the primary Si and the secondary *α*-Al. The corresponding nucleation temperatures of the Si and *α*-Al phases for Particle I cooled at 5000 K/s are 627 °C and 521 °C, respectively. Note that the nucleation temperature of the *α*-Al + Si eutectics cannot be evaluated precisely because of the overlapping solidification peaks (at ca. 500 °C) between *α*-Al dendrites and *α*-Al + Si eutectics, as shown in Figure 3. For the nucleation of the primary Si, the nucleation undercooling can be evaluated by Equation (1) with the liquidus temperature of the Al-20Si alloy (684 °C), which is 57 K. After the nucleation of primary Si, primary Si grows and rejects Al into the melt with decreasing temperature. At 521 °C (lower than the eutectic temperature 577 °C [24]), Al nucleates on the interface of primary Si and starts to grow in the melt. The melt is depleted of Si because of the solidification of primary Si. Assuming that the concentration of Si in the melt in front of the solidifying Si follows the metastable extension of the Al liquidus line in the Al-Si phase diagram [24] during the rapid solidification of primary Si, the concentration of Si is 9% (mass%) at 521 °C. The liquidus temperature at this concentration is 597 °C [24], and thus, the nucleation undercooling of *α*-Al is 76 K.

**Figure 4:**
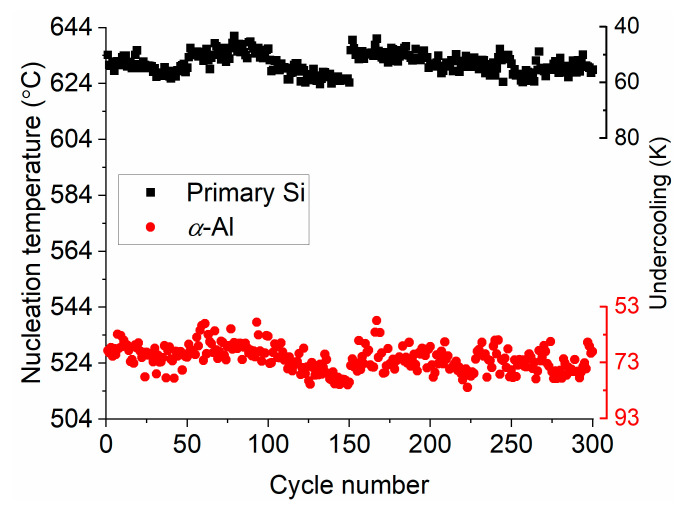
Nucleation temperatures and undercoolings of primary Si and α-Al dendrites as measured from 300 cycles.

For a sound statistical basis, another particle (Particle II) with the same size (25 ± 0.5 μm in diameter) as Particle I was heated and cooled at 5000 K/s for 300 times. Figure 4 shows the nucleation temperatures and undercoolings of the Si and *α*-Al phases for each of the 300 nucleations in chronological order. Both nucleations occur in bands of approximately 30 K width because of the stochastic nature of nucleation. The nucleation temperatures of the primary Si and *α*-Al for Particle I are also in these bands.

**Figure 5:**
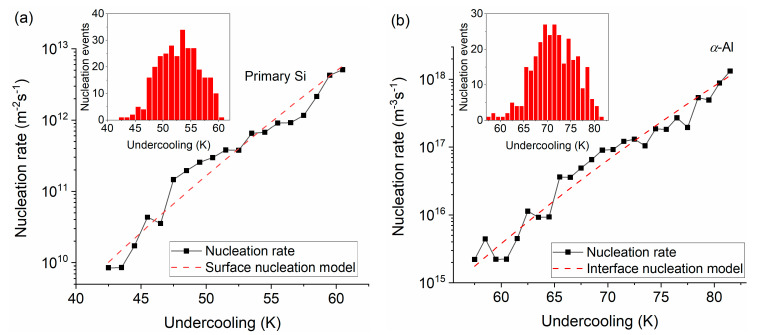
Nucleation rates as a function of undercooling for the (**a**) primary Si and (**b**) α-Al dendrites. The dashed curves indicate the best fit to the data for the surface and interface heterogeneous nucleation models. Distributions of nucleation events versus undercooling are shown in the insets.

If nucleation events are independent and occur randomly in time as described in CNT, the events fulfill the requirements for a Poisson process. These requirements can be fulfilled for the nucleation process of single metallic particles [16,27,28]. Assuming one nucleus forms in one nucleation event at the solidification onset temperature, the nucleation rate per unit volume and unit surface area, *J_V_* and *J_S_*, can be expressed as [29,30]:

(2) JS=λβS

and:

(3) JV=λβV

where *V* and *S* are the volume and surface area of the melt, *β* is the cooling rate, and *λ* is the time-dependent rate constant of an inhomogeneous Poisson distribution, which can be determined from the histogram distributions of Si and *α*-Al (see the insets of Figure 5) using the following equation [27]:

(4) $$\lambda =\frac{a}{b\left(s+a/2\right)}$$

where *b* is the width of the temperature interval (=1 K in this study). In one of the temperature intervals, *a* nucleation events occur and *s* nucleation events remain molten to enter the next temperature interval. As mentioned above, the surface nuclei of the Si phase form at the surface of the particle (*S* = *πd*^2^, 1963 ± 80 μm^2^, where *d* is the particle diameter), and the nucleation rate of the primary Si is calculated by Equation (2), as shown in Figure 5a.

After solidification of the primary Si, the volume of the melt is reduced and the residual Si in the melt is less than the initial melt concentration. Hence, the volume of the melt and the amount of residual Si in the melt should be estimated. In the absence of the solidification enthalpy of Si in the deep undercooled region, the melting enthalpy of Si is used as its solidification enthalpy at ca. 627 °C. Thus, the mass of the solidified Si, *m_Si_*, is calculated by the following expression:

(5) $$m_{Si}=\frac{\Delta H_{s\_DFSC}}{\Delta H_{m\_Si}}$$

where Δ*H_s_DFSC_* is the solidification enthalpy measured by DFSC (2.4 ± 0.2 μJ for the DFSC curve shown in Figure 3) and Δ*H_m_Si_* is the melting enthalpy of Si (1726 J/g) [31]. It should be pointed out that Δ*H_s_DFSC_* was evaluated by integrating the Si solidification shoulder (peak) until *α*-Al starts to solidify, as shown in Figure 3. It is assumed that the solidification of Si is limited by the nucleation events of *α*-Al. Thus, the mass of the solidified Si, *m_Si_s_*, can be calculated and equals 1.4 ± 0.1 ng, corresponding to a volume of 600 ± 40 μm^3^. Then, assuming that the nuclei for *α*-Al form on the primary Si (interface nucleation on the primary Si), whose volume is 7581 ± 480 μm^3^, the nucleation rate for *α*-Al is calculated by Equation (3), as shown in Figure 5b. As expected, both nucleation rates increase with increasing undercooling. It is supposed that the size of the primary Si and *α*-Al decrease with increasing undercooling.

To understand the tendency between the nucleation rate and undercooling in terms of the spherical cap model of CNT, the expressions for the surface and interface heterogeneous nucleation rates of a single particle during rapid solidification are derived in [32,33] as:

(6) $$J_{S}=n_{S}\frac{D_{l}}{a_{0}}\sqrt{\frac{\sigma_{ls}}{k\left(T_{m\_onset}-\Delta T\right)}}\exp\left(-\frac{\Delta G^{*}f_{S}}{k\left(T_{m\_onset}-\Delta T\right)}\right)$$

and:

(7) $$J_{V}=n_{V}\frac{D_{l}}{a_{0}}\sqrt{\frac{\sigma_{ls}}{k\left(T_{m\_onset}-\Delta T\right)}}\exp\left(-\frac{\Delta G^{*}f_{V}}{k\left(T_{m\_onset}-\Delta T\right)}\right)$$

with:

(8) $$\Delta G^{*}=\frac{16\pi \sigma_{ls}^{3}}{3\Delta G_{V}^{2}}$$

where *n_S_* and *n_V_* are the densities of surface and interface heterogeneous nucleation sites (the numbers of potential surface and interface heterogeneous nucleation sites per unit surface area and unit volume), respectively, *D_l_* is the liquid diffusivity, *a*_0_ is the atomic spacing, *σ_ls_* is the solid-liquid interfacial energy (surface tension), *k* is the Boltzmann constant, *f_S_* and *f_V_* are the shape factors for surface and interface heterogeneous nucleation, and ∆*G_V_* is the free energy difference between the melt and the solidified phase. The shape factors describe the potency of the heterogeneous nuclei.

During the DFSC measurements, an inherent Al_2_O_3_ layer always exists, while the primary Si is solidified from the surface of the particle, as shown in Figure 2. Therefore, it is assumed that the surface nuclei for Si form in a spherical cavity at the surface of the particle (surface nucleation on the Al_2_O_3_ oxide layer). For simplicity of the model, it is also assumed that the interface nuclei for *α*-Al form in a spherical cavity (at a nanosized scale) inside the particle (interface nucleation on the primary Si). Thus, the shape factors, *f_S_* and *f_V_* is given by [34]:

(9) $$f_{S,V}=\frac{1}{4\rho_{S,V}^{3}}\left(-1+2\rho_{S,V}+\sqrt{1+\rho_{S,V}^{2}+2\rho_{S,V}\cos\theta_{S,V}}\right){\left(1+\rho_{S,V}-\sqrt{1+\rho_{S,V}^{2}+2\rho_{S,V}\cos\theta_{S,V}}\right)}^{2}$$

with:

(10) $$\rho_{S,V}=\frac{r^{*}}{R_{S,V}}$$

where subscript *_S,V_* designates the surface and interface heterogeneous nucleation, respectively, *θ_S,V_* is the contact angle between the heterogeneous nucleus and the undercooled melt, *R_S,V_* is the radius of the spherical cavity, and r^*^ is the critical radius of a nucleus. Based on CNT, r^*^ is given by:

(11) $$r^{*}=\frac{2\sigma_{ls}}{\Delta G_{V}}$$

Due to the lack of specific heat capacity data for each phase, especially in the undercooled region, ∆*G_V_* is estimated by a parallel tangent construction [35]. Based on a thermodynamic assessment of the Al-Si system, the Gibbs energies of solid Al, solid Si, and liquid phases are represented by polynomial expansions [24]:

(12) Gi=G0Ali(1−x)+G0Siix+RT[xlnx+(1−x)ln(1−x)]+x(1−x)[Ai+Bi(1−2x)+Ci(1−6x+6x2)]

where *i* designates the phase, *x* is the atomic fraction of Si (0.19 and 0.09 for the nucleation of Si and Al, respectively), *R* is the gas constant, *T* is absolute temperature, G0Ali and G0Sii are the lattice stability terms of the pure Al and Si, and *A^i^*, *B^i^*, and *C^i^* are the interaction parameters that are linearly dependent on temperature. Table 2 lists the parameters for Equation (12).

The development of a complete description of the density of nucleation sites within an undercooled melt has been a continuing challenge. As mentioned above, the primary Si nuclei form on the surface of the particle. Therefore, it is assumed that the surface heterogeneous nucleation of Si could occur on the whole Al_2_O_3_ layer on the surface of the Al-20Si particle. Thus, the density of surface heterogeneous nucleation sites for primary Si can be expressed by [23]:

(13) $$n_{S}=\frac{N_{A}h}{V_{m}}$$

where *N_A_* is the Avogadro constant, *h* is the thickness of the Al_2_O_3_ layer (=1 nm) [36], and *V_m_* is the molar volume of Al_2_O_3_ (=25.7 × 10^−6^ m^3^/mol) [36]. Thus, *n_S_* equals 2.343 × 10^19^ m^−2^. For the interface nucleation of *α*-Al, the density of interface heterogeneous nucleation sites in the melt is given by [15]:

(14) $$n_{V}=n_{V}^{0}\exp\left(c\Delta T\right)$$

where $n_{V}^{0}$ is the base level of nucleation sites (=10^13^ m^−3^) and *c* is a positive constant (=0.059) [15].

Based on this model (Equations (6)–(14)), the relationship between the nucleation rate and nucleation undercooling for the rapid solidification of single particles with a certain heterogeneity can be estimated. Employing the obtained parameters listed in Table 3, the data were fitted for Si and *α*-Al phases. Here *θ_S_*, *θ_V_*, *R_S_*, and *R_V_* are fitting parameters. Levenberg-Marquardt nonlinear least-squares curve fitting was utilized with boundary conditions of 20° < *θ_S_*_,*V*_ ≤ 180° and 0.1 nm < *R_S,V_* < 10 nm. For the spherical cap model, the contact angle is larger than 20° [37]. The fitted curves are indicated by dashed lines in Figure 5. The best fits to the data yield *R_S_* = 1.29 nm and *θ_S_* = 74.6° for the surface nucleation of Si, and *R_V_* = 2.61 nm and *θ_V_* = 53.4° for the interface nucleation of *α*-Al. As shown in Figure 5, the modeling of the nucleation rates based on CNT with the inhomogeneous Poisson analysis of the thermal cycling behavior gives an accurate description of the 300 nucleation measurements for the single micro-sized Al-20Si particle that is rapidly solidified at 5000 K/s. Moreover, the nucleation mechanisms for the primary Si and secondary *α*-Al are well described as surface and interface heterogeneous nucleation in a spherical cavity.

## 4. Conclusions

In-situ DFSC was successfully used to investigate the rapid solidification process of a single micro-sized Al-20Si (mass%) particle at a cooling rate of 5000 K/s. By DFSC treatments at the same cooling rate, nucleation undercooling of one single micro-sized particle was correlated with the microstructure analysis of another similarly particle (with the same size). The results show that the microstructure of the rapidly solidified particle consists of primary Si, secondary *α*-Al, and (*α*-Al + Si) eutectics.

The nucleation rates of the primary Si and *α*-Al dendrites were obtained by a statistical analysis of 300 solidification events (300 heating–cooling cycles of one single particle by DFSC). According to a modified classical heterogeneous nucleation theory, two different nucleation mechanisms of the primary Si and secondary *α*-Al were proposed, i.e., surface heterogeneous nucleation for Si and interface heterogeneous nucleation for *α*-Al.

By controlled rapid cooling and known nucleation undercooling via in-situ DFSC with microstructure characterization, it is possible to investigate the rapidly solidified structures and the nucleation mechanisms of single metallic particles. Therefore, this is a practical approach for a detailed investigation of the rapid solidification processes of metal particles and to discriminate surface and interface nucleation.

## Figures and Tables

**Table 1:** Mass fractions of alloying elements in the gas-atomized Al-20Si powder particles.

Alloy	Si	Fe	Mg	Ag	Ti	Cu	Al
Al-20Si	20%	0.27%	0.07%	0.03%	0.02%	0.02%	Bal.

**Table 2:** Thermodynamic model parameters, J/mol, T in K [15,24].

G0Alfcc=−10,792+11.56T	$A^{l}=-10,695.4-1.823T$
G0Sifcc=12.12T	$B^{l}=-4275.5+3.044T$
G0Sidia=−50,600+30.00T	$C^{l}=670.7-0.460T$
G0Aldia=30.00T	$A^{fcc}=-200-7.594T$
G0All=0	$A^{dia}=89,138-31.445T$
G0Sil=0	

**Table 3:** Parameters used for nucleation rate analysis.

Parameters	Value
Liquidus temperature (Si), *T_l_^Si^*	684 °C [24]
Liquidus temperature (Al), *T_l_^Al^*	597 °C [24]
Liquid diffusivity, *D_l_*	5 × 10^−9^ m^2^/s [38]
Interfacial energy (Al in Al-Si system), *σ_sl_^Al^*	0.169 J/m^2^ [39]
Interfacial energy (Si in Al-Si system), *σ_sl_^Si^*	0.352 J/m^2^ [39]
Atomic spacing (Al), *a*_0_*^Al^*	2.6 × 10^−10^ m (pure Al) [27]
Atomic spacing (Si), *a*_0_*^Si^*	3.0 × 10^−10^ m (Si atomic diameter) [40]

## Data Availability

The datasets generated during and/or analyzed during the current study are available from the corresponding author on reasonable request.
